# “It’s Pure Chaos Every Day”: COVID-19 and the work of Canadian federal institutional parole officers

**DOI:** 10.1177/20662203211056487

**Published:** 2022-04

**Authors:** Mark Norman, Rosemary Ricciardelli

**Affiliations:** 3710 McMaster University, Hamilton, ON, Canada; 7512 Memorial University of Newfoundland, St. John’s, NL, Canada

**Keywords:** COVID-19, parole, prison, risk, stress

## Abstract

As the Canadian federal correctional system grappled with the onset of the COVID-19 pandemic, institutional parole officers, who play a central role in prisoners’ case management team, remained essential service providers. Working in uncertain circumstances, these correctional workers navigated new and rapidly changing protocols and risks, while attempting to continue to provide support to those on their caseloads. Based on semi-structured interviews with 96 institutional parole officers, conducted after Canada’s “first wave” of COVID-19 infections, we analyze three ways in which their work was impacted by the pandemic: shifting workloads, routines, and responsibilities; increased workloads due to decarceration (i.e., efforts to reduce the number of incarcerated individuals); and the navigation of new forms of risk and uncertainty. This study advances the understanding of stress and risk in probation and parole work and presents recommendations to ameliorate the occupational stresses experienced by correctional workers during and beyond COVID-19.

## Introduction

Correctional Service of Canada (CSC) employs over 1200 parole officers, who either work in one of the 43 federal correctional institutions or at parole offices or residential facilities in the community (CSC, 2019a). CSC is responsible for the incarceration and community supervision of individuals given sentences of 2 years or more, while provincial and territorial correctional systems are responsible for people whose sentences are a maximum of 2 years less a day. A case management team, assigned to each federal prisoner, is responsible for developing a correctional plan outlining “treatment and recommendations for rehabilitation… [and] serves as a basis to monitor the inmate’s progress throughout their sentence” (CSC, 2019b, *Discussion*). CSC’s institutional parole officers (IPOs), typically supervising caseloads of between 24–32 prisoners, play a central role in developing and monitoring prisoners’ progress against their correctional plan. IPOs hold regular meetings with prisoners on their caseload, help them access programs that contribute to their correctional plan, work with a community parole officer to develop a supportive release plan, and ultimately make recommendations to the Parole Board of Canada about whether or not a prisoner should be released (CSC, 2019b). Most prisoners can apply for full parole once they have served the earliest of 7 years or one-third of their sentence (prisoners with life sentences face longer eligibility periods), and may apply for day parole in advance of their application for full parole. All prisoners, except those with indeterminate or life sentences, are granted statutory release for the final one-third of their sentence, unless the Parole Board of Canada issues a detention order to keep the prisoner incarcerated for the remainder of their sentence (CSC, 2019c). Prisoners may also, in rare instances such as terminal illness, be granted parole by exception—referring to extraordinary circumstances motivating a need for accelerated parole (Parole Board of Canada, 2021).

Like other correctional systems around the world, the Canadian federal correctional system was significantly impacted by the COVID-19 global pandemic, which was declared by the World Health Organization in March, 2020. As of 12 April 2021, 29 federal correctional institutions had reported outbreaks, 1463 prisoners had tested positive for COVID-19, and 5 had died (CSC, 2021). As of 23 February 2021, the rate of federal prisoner infection was higher than 10%, over five times that of the general Canadian population (Office of the Correctional Investigator, 2021). Further, as of 8 February 2021,140 CSC employees had tested positive for COVID-19 (Public Safety Canada 2021). Within the first month of the pandemic, Canada’s Minister of Public Safety requested that the CSC and Parole Board of Canada “consider early release for some federal inmates to mitigate the impact of COVID-19 behind bars” (Harris, 2020, para. 1). The push for decarceration—which can be defined as “alternatives to incarceration, such as serving sentences in the community rather than in prison, as well as the premature conclusion of a criminal sentence, and the aggregate reduction in the prison population” (Ricciardelli et al., 2021, p. 495, p. 495)—was part of a broader global effort to reduce the impact of COVID-19 among prisoners, particularly those advanced in age, or with underlying health conditions or other physical vulnerabilities (Burki, 2020). However, in the Canadian federal system, COVID-19 had minimal impacts on decarceration (Ricciardelli et al., 2021). The Parole Board of Canada (2021) explains that between March, 2020 and April 2021, “13 parole by exception cases have been granted and 9 are pending decision, compared to only 7 parole by exception cases for all of last fiscal year, of which 4 were granted.”

In the current article, we explore how the COVID-19 pandemic affected Canadian IPOs’, who remain essential service providers (and thus working through lockdowns), workplace stress and perceptions of occupational risk. As part of their daily work, IPOs assess and manage a variety of occupational risks and experience diverse stressors, some operational and others organizational (Norman and Ricciardelli, 2021). IPOs do so in an often stressful work environment where the possibility of exposure to potentially psychologically traumatic events, including vicarious trauma (e.g., victim statements, graphic images, etc.), is the norm rather than the exception (Page and Robertson, 2021; Rhineberger-Dunn et al., 2016). The onset of the COVID-19 pandemic both exacerbated existing, and created new forms of, occupational risk and stress that IPOs were forced to navigate. Working in the uncertain circumstances created by the pandemic, IPOs navigated new and rapidly changing protocols and risks, while attempting to continue to provide support to those on their caseloads. Based on semi-structured interviews with 96 IPOs working for the CSC, in the current article we analyze how the “first wave” of the pandemic affected their workloads, responsibilities, and their understanding and navigation of their occupational risks. We know relatively little about how parole officers (POs) understand and navigate these occupational challenges. As such, via the current study, we advance the understanding of stress and risk in parole work and provide a foundation for evidence-based recommendations to ameliorate the occupational stresses experienced by POs and correctional workers beyond COVID-19.

### COVID-19 and prisons

At the onset of the COVID-19 pandemic, researchers and health professionals expressed concern about how the virus would affect people living in congregate settings, such as long-term care facilities (Lai et al., 2020) and shelters (Tsai and Wilson, 2020). Like these spaces, prisons were identified as high-risk sites for rapid contagion due to intersecting factors that included the health and social vulnerability of their residents, (over)crowded living arrangements that made physical distancing difficult or impossible, shared dining and sanitation spaces (e.g., bathrooms and showers), and the daily movement of large numbers of people (i.e., staff and visitors) into and out of prisons (Barnert et al., 2020; Kinner et al., 2020; Ricciardelli et al., 2021; Yang and Thompson, 2020). In response to the challenges posed by COVID-19, prison systems internationally adopted various risk mitigation strategies, including mandating masks or personal protective equipment (PPE) for staff, regularly testing staff and prisoners, limiting prisoners’ time outside their cells, eliminating visits and programming, reducing staffing levels, and reducing the prisoner population (Brennan, 2020; Lindström et al., 2020; Marcum, 2020; Ricciardelli et al., 2021).

While researchers have focused on the potential negative impacts of COVID-19 on prisoners—including both the risk of contagion and the mental and social health toll of increased restrictions on movement, visits, and access to programs and recreation opportunities (e.g., Ricciardelli et al., 2021; Ryan et al., 2020; Stewart et al., 2020)—fewer have considered the experiences of prison staff during the pandemic. Researchers have found that prison workers may face heightened risk of acquiring COVID-19 compared with the general population (Nowotny et al., 2021) or experience psychological distress arising from increased risks and the demanding nature of prison work in the midst of a pandemic (Kothari et al., 2020; Testoni et al., 2021). Given that “staff and officers must balance staying safe and healthy while maintaining the care, custody, and control of incarcerated persons as well as striving not to infect their family members and friends or bringing COVID-19 into the institution” (Ricciardelli et al., 2021: 491), there is a need for more investigation of prison workers’ experiences during the COVID-19 pandemic.

### Stress and risk in correctional work

Scholars recognize that PO stress emerges from a variety of sources, including personal circumstances, job content, and factors that are external or intrinsic to the organization (Slate and Johnson, 2013). Following studies on stressors in policing work (Duxbury et al., 2015; Ricciardelli, 2018), Norman and Ricciardelli (2021) categorized stressors faced by probation and parole officers (PPOs) in their jobs as either operational (i.e., inherent to the duties of the job) or organizational (i.e., arising from characteristics of the organization itself). Within this framework, the operational stressors PPOs experience include perceptions or risk to personal safety (Finn and Kuck 2005; Lewis et al. 2013) and exposure to potentially difficult materials and the resultant potential for experiences of secondary trauma (Norman and Ricciardelli 2021; Page and Robertson, 2021; Rhineberger-Dunn et al., 2016; Westaby et al., 2016). Meanwhile, common organizational stressors identified by researchers of PPOs include high workload volumes (e.g., paperwork or other administrative tasks), large caseload sizes, understaffing or lack of staff support, and tensions with colleagues or management (DeMichele and Payne, 2007; Farrow, 2004; Finn and Kuck, 2005; Norman and Ricciardelli 2021; O’Donnell and Stephens, 2001; Phillips et al., 2016; Slate and Johnson, 2013). The cumulative impact of occupational stressors can harm PPOs’ work–life balance, forcing them to manage the “spillover” of work into their personal lives (Westaby et al., 2016). In contrast to the sizeable literature on PPO stress, scant literature explores how PPOs perceive and navigate risk in their work. Rather, researchers considering risk in PPO work have largely focused on risk assessments of parolees/probationers (e.g., Persson and Svensson 2011; Weaver and Barry 2014).

While parole work is typically understood as occurring in the community (Page and Robertson, 2021), the majority of the day-to-day work of CSC IPOs takes place within correctional institutions. As such, IPOs may experience additional operational stressors arising from the nature of prison work. Researchers, mostly focused on correctional officers (COs), have shown that prison workers may experience operational stressors such as personal safety fears (Arnold, 2017; Lambert and Hogan, 2018; Ricciardelli, 2019) and work–family conflict that are exacerbated by immersion in a prison (Armstrong et al., 2015; Lambert and Hogan, 2018). Researchers investigating the mental health of Canadian public safety personnel found that correctional workers face high levels of exposure to potentially psychologically traumatic events, such as experiencing or witnessing physical violence (Carleton et al., 2019), yet also noted the “important role of non-traumatic work-related [organizational and operational] stressors on the psychological health” of Canadian public safety personnel (Carleton et al., 2020: 19). Although IPOs do not share the same security and first responder responsibilities as COs (Ricciardelli, 2019), they do work in the same institutional environment and share some of the same risks and occupational stressors. The current study adds to the developing scholarly understanding of how the COVID-19 pandemic has impacted the work of prison staff and POs, by examining the experiences of Canadian IPOs working in an institutional setting. No researchers have specifically studied the work experience of IPOs, including during COVID-19.

## Methods

The current study was commissioned by the Union for Safety and Justice Employees (USJE), which represents federal parole officers, and was approved by the research ethics board at Memorial University of Newfoundland (#20201495). CSC assisted with recruitment by distributing study documents in both French and English to parole officers through internal email listservs. In total, for the current study, we interviewed 96 participants with experience as IPOs, who are part of a larger sample of 150 federal parole officers (54 were community parole officers). Federal government organizations, including CSC, operate in Canada’s two official languages: English and French. As such, recruitment materials were provided in both languages. Most IPO participants (*n =* 93) participated in one-on-one telephone interviews in English, the language in which the researchers are fluent. To ensure the inclusion of Francophone participants, we also offered two French language focus groups, which were professionally translated in real-time. Due to the cost of hiring translators and the difficulty of coordinating participant schedules, the focus groups included both community (*n =* 2) and institutional (*n =* 3) parole officers; while the discussions often focused on commonalities between these roles, the interviewer ensured that participants could speak about unique aspects of their position (e.g., working in a prison environment).

The majority of participants (91.7%; *n =* 88) were working as IPOs at the time of the interview, while the remainder (8.3%; *n =* 8) were employed in temporary or permanent managerial positions but had previously worked as IPOs. Participants’ occupational tenure with CSC ranged from one to 30 years; with a median of 13 years. Our sample included IPOs working in all eight Canadian provinces that have a CSC institution (i.e., Ontario (32.3%; *n =* 31), British Columbia (28.1%; *n =* 27), Alberta (11.5%; *n =* 11), Quebec (9.4%; *n =* 9), Manitoba (9.4%; *n =* 9), Saskatchewan (5.2%; *n =* 5), New Brunswick (2.1%; *n =* 2), and Nova Scotia (2.1%; *n =* 2)). Most participants identified as female (78%; *n =* 75), with 19.8% (*n =* 19) identifying as male and 2.1% (*n =* 2) not identifying their gender. Most participants identified as white (89.6%; *n =* 86), with seven individual participants identifying as either Black, Canadian, Chinese, Indigenous, Korean, Latin American, or South Asian (*n =* 1; 1.0% each); three participants (3.1%) refrained from answering. In total, 39 (40.6%) participants were between 35–44 years old, 27 (28.1%) were between 45–54, 15 (15.6%) were between 25–34, eight were between 55–64 (8.3%), three were between 19–24 (3.1%), two were between 65–74 (2.1%) and two did not provide their age (2.1%).

Interviews were semi-structured, allowing participants to guide the conversation, and share experiences or identify issues that they felt were most relevant. Most interviews were approximately one to 2 hours in length and, due to geographic limitations and COVID-19 restrictions, we conducted the interviews on the phone. Although face-to-face interviews are predominant in qualitative research, there is evidence that telephone interviews do not inhibit rapport-building and may permit participants to discuss sensitive topics with greater comfort (Mealer and Jones, 2014; Novick, 2008); the latter benefit of telephone interviews was particularly salient for our study, given that participants regularly discussed difficult or potentially psychologically traumatic occupational experiences. Interviews were conducted between August and October 2020. Regarding COVID-19, the time period was after Canada’s “first wave” of COVID-19 infections, which peaked at close to 2760 new cases per day nationwide in early May (CBC News, 2021). Data collection ended as cases were beginning to climb toward a much more significant “second wave,” which ultimately peaked in January 2021. As such, our data are limited, given in discussing the impact of COVID-19 on their work and workplaces, participants were reflecting on the initial impact of the pandemic, without knowledge of the extent of the increased rates of transmission given the immergence of variants that would eventually occur.

Interviews were transcribed verbatim and transcripts were coded in an open-ended fashion to determine emergent themes, following a semi-grounded constructed approach (Charmaz, 2014; Glaser and Strauss, 1967; Ricciardelli et al., 2010). An initial set of codes was developed by the researchers, who independently and sequentially coded five transcripts to ensure inter-rater reliability. The remaining transcripts were then coded by the research team, allowing the initial codes to be refined and new ones to be created as they emerged from the data. QRS NVivo Pro was used to facilitate autocoding and to assist researchers in coding data into primary, secondary, and tertiary themes. Axial coding was employed to connect and develop these themes (Strauss and Corbin, 1990).

## Results

In our analysis of IPOs’ discussions of COVID-19, 3 dominant themes emerged: occupational changes (e.g., shifting workloads, routines, and responsibilities); the impact of decarceration on workloads; and the navigation of new forms of risk and uncertainty. In unpacking participants’ experiences, we note that COVID-19 had significant impacts on both operational and organizational stressors for IPOs at the CSC. Further, while our focus is on the occupational experiences of IPOs, many participants expressed the view that the challenges faced by IPOs during the COVID-19 pandemic were also having detrimental impacts on the prisoners under their supervision.

### Changing workloads, routines, and responsibilities

Participants consistently described COVID-19 as having caused upheaval to their daily work routines. CSC typically expected IPOs to be on-site at the institution every day prior to the pandemic and now they were working partially or fully from home. Participants described variation in the extent of telework and the amount of time they spent at the institution. Participant 2, for example, expresses a typical disruption to his work routine:

We’ve gone from a system where we would go in five days a week, Monday through Friday, to working remotely the vast majority of the time. We were told to go down to 30 percent of our case management staff in on any given weekday, so we developed a schedule where we working [on-site] a maximum of two days a week.

Participant 2 describes how, at his place of work, on-site staffing levels were reduced as part of an institutional risk management strategy designed to minimize close physical proximity and, thus, to reduce the possibility of COVID-19 transmission. The blend of on-site work and telework described by participant 2 had mixed impacts on the workload of IPOs. For some participants, working from home afforded greater flexibility that enabled them to complete their work more efficiently. Participants explained that particular tasks, most notably report writing, were easier to focus on away from the chaotic work environment of a prison:

Working from home there’s less interruptions than when you’re at work, so you get ahead of the game. Because you might only get one report done at work, ’cause you’ve got a meeting or you’re on the phone or an inmate comes to see you. Whereas I might get two or three reports done when I’m at home ’cause I’ve got nothing distracting me. (participant 110)

Participant 110’s words demonstrate how, for some participants, telework enabled them to compartmentalize their work toward being more productive. However, despite the benefits of telework for completing specific job tasks, IPOs recognized that face-to-face interaction with colleagues or prisoners were necessary for effective case management. Many participants found that work-from-home arrangements allowed for a balance to be struck between these responsibilities:

You need to be available. You need to be meeting with inmates…. But there’s also times where we need quiet time to be able to write reports and not be interrupted. ’Cause we do face a lot of interruptions in that [prison] environment. (participant 38)

While participants 110 and 38 highlight positive changes to work routines arising from COVID-19, particularly uninterrupted report writing time, many other IPOs described an increased workload and stress resulting from the pandemic work arrangements. Participants expressed that their ability to separate their professional and personal lives suffered while working the majority of their time at home, as their job duties bled into their personal time and spaces:

I tend to work beyond my work hours and on weekends because it’s available to me. So there’s no disconnect anymore. Whereas before I could leave my office and I could kind of like destress on my drive home, now it’s just like continuous, it’s always a part of my life. So that’s been a challenge. (participant 20)

In addition to the work–life balance impact described by participant 20, working from home made meeting the prisoners on their caseload challenging. Meetings were by phone or, irregularly, in-person, which adversely affected their ability to monitor their clients’ adherence to their correctional plans and assess their risks and needs. IPOs who felt that in-person interactions were essential for forming their professional assessments felt the greatest impact:

Being somebody that likes that one-on-one intervention with the offenders, like meeting with them physically and talking about things. I don’t feel comfortable writing reports that essentially control somebody else’s life without them having the ability to provide input that I can take into consideration. It’s just how I am…. I just like to have those follow up with them and stuff like that. It helps in rapport building, and that simultaneously helps in public safety and in some way shape or form cause they do build trust a lot. (participant 28)

Participant 28’s words show the barriers to engagement created by the pandemic were not merely inconveniences for IPOs—they also had potential consequences for prisoners. IPOs’ recommendations influence each prisoner on their caseload’s likelihood of earning parole, receiving a transfer to a lower security level, or accessing a program, and so on. As such, the challenges faced by IPOs during the pandemic also had potentially damaging implications for the prisoners on their caseloads—another source of stress for IPOs. Furthermore, many IPOs expressed genuine concern about the mental and physical health of prisoners, who were enduring greater-than-normal restrictions (including lengthy lockdowns) and uncertainty or fear about potential exposure to COVID-19.

Staffing levels were also identified as a major source of stress by many participants. There was reduced numbers of IPOs on-site at a given time, and program officers—who are responsible for providing prisoners with social, educational, occupational, and recreational programs that are intended to contribute to their overall correctional plan—were ordered to stay home. In these circumstances, IPOs felt that they had to shoulder increased responsibilities with inadequate resources or knowledge. Participant 40 explained:

I would say the staff [drove the increase in workload]. Like a lot of staff going home. If I take, for example, the correctional programs officers being sent home: a lot of the times, the guys will be in programs and we’ll be able to kind of manage their stressors throughout the interventions that are done with programs. So, then a lot of the stuff that maybe usually a program officer would have dealt with [prior to COVID-19], then the parole officers had to deal with…. I found that parole officers, at our site anyways, tend to be kind of like the dumping ground.

Participant 40’s description of being a “dumping ground” suggests that staffing decisions made in response to the pandemic, like program officers being away from the institution, exacerbated existing sources of stress and left IPOs to handle job duties and prisoner expectations that were usually shared with other staff members. Recall, IPOs were one the few supports still available to incarcerated individuals during COVID-19. Asked what the most challenging aspect of the pandemic has been, participant 104 elaborated on how reduced staffing created stressful work conditions:

I would say just the chaos of it all, really. Because you know when you go in on any given day, there’s one team of you there, so whatever has to be done, has to be done. It probably isn’t your case, so you have all this stuff that needs to be done, but you don’t really know the case. So you’re kind of scrambling trying to figure things out, trying to figure out whatever needs to be done. And we all do our best to kind of help each other out, but it’s just it’s pure chaos every day.

In the above excerpt, participant 104 notes an additional challenge posed by reduced staffing during the pandemic: that the rotational schedule meant that the IPOs on-site had to work with prisoners who were not usually on their caseload, and thus handle cases with which they felt unfamiliar. Like other IPOs, participant 104 found the pandemic-induced staffing levels to be an added source of stress and, thus, described her work as “pure chaos every day.”

### Decarceration

Many participants reported that calls for decarceration affected their workloads. IPOs explained, in the context of reducing the potential footprint of COVID-19 in prisons and media reports the federal government was seeking to reduce the population in CSC correctional institutions, they faced a higher-than-usual volume of applications for release from prisoners. Although most applications were unlikely to be supported by the IPO or granted by the Parole Board of Canada, IPOs were still required to undertake the work to fulfill prisoners’ requests. Statements by two IPOs illustrate this state of affairs:

I had several inmates put in [for parole]. I actually have a guy that I’ve been dealing with right now, I was talking to yesterday. He readily admits he got caught up in this sweeping emotion in the inmate population that you should try and get parole, [that] with COVID they’ll let you out, they’ll let you out. And he didn’t intend on applying for parole that quickly but he kind of got swept up in the emotion at the time and he put it in. So I’ve had a few guys like that, I had one guy apply for parole by exception, so I had to do that paper work as well. I mean, today, none of them have been successful. (participant 44)

My workload increased drastically, I had a lot of guys applying for parole thinking that this is the get out of jail free card…. I was looking at the number of reports that I’ve done in this timeframe and its double what I did last year at this timeframe. (participant 99)

As the statements of participant 44 and 99 indicate, many IPOs experienced increased workloads due to a spike in prisoner applications for parole. For some IPOs, prisoners seeking parole by exception, a rare form of early release intended for prisoners “who are terminally ill or whose physical or mental health is likely to suffer damage if the offender continues to be held in confinement” (PBC, 2021, “Safe Release of Offenders”), created additional stresses on their workload. Speaking directly to applications for parole by exception, Participant 44 describes the challenge:

Parole by exception is not something that I dealt with before, so that was an extra, application with extra work and then it was very time sensitive as well. And so you’re doing everything. Like, we have our [normal] timeframe set out by policy, and then all of a sudden parole by exception comes along and all those timeframes go out the window and everything needs to be done as soon as possible. And, like I said, I had guys that we had a plan, as far as when they would apply for parole, but they jumped the gun [and] applied earlier because of COVID…. The work would have been coming one way or another, but it came earlier than expected.

Participant 44’s statement captures the workload challenges posed by an unexpected rise in parole by exception requests, which forced IPOs to navigate a time-sensitive process with which they were un- or not very familiar. The requests added learning about an application process in addition to creating additional workload stresses with critical deadlines. An additional consequence of applications for parole by exception for IPOs was the need to make professional risk assessments they felt were beyond their remit. Exacerbating the challenge was inaccessible information on the health risks of potential parolees, yet IPOs felt they were still expected to weigh their risk to public safety against their health risks due to COVID-19. Participant 3 explained:

Information we got about guys who are high risk though was really sketchy and unclear. Again, we were sort of told ‘so these are the guys on your caseload that are high risk, so you can take a look at them perhaps for a different type of release,’ but we weren’t told what’s wrong with them. So I don’t know, does he have asthma? Or does he have leukemia? Or, I just don’t know?… Even when the information to given to me I’m still like ‘I don’t know, does that mean, like how high a risk is he?’ Is he a is he a such a high risk for death that it overshadows his risk to hurt someone if I support [his exceptional release]?... It’s, like, their health, their future, as well as public safety.

As Participants 44 and 3’s words demonstrate, the COVID-19 pandemic created conditions that forced some IPOs to accommodate an unusually high number of applications for exceptional release, to process them within a tight timeframe, and to make risk assessments that they did not feel qualified to determine. IPOs faced these challenges while also managing their normal caseloads in the circumstances created by the pandemic (and managing clients outside their caseload when on-site). Further, despite the impression of some prisoners that COVID-19 would increase their likelihood to attain early release, the calls for decarceration in federal correctional institutions had minimal impact on the number of exceptional releases, leaving IPOs frustrated at spending time on these unfruitful applications.

### New forms of risk and uncertainty

IPOs expressed concern about inadequate safety protocols inside institutions, which left them feeling vulnerable to exposure to COVID-19 in the first wave of the pandemic. Specific complaints included a lack of materials, such as masks, PPE, or disinfecting wipes. Participant 3 exemplified these concerns, stating:

I went on this huge tear a few weeks ago. I wanted to get every parole officer issued a couple of extra masks and a thing of wipes. Because, you know, we might grab a boardroom with a guy that hasn’t been cleaned in who knows how long. And I couldn’t get it. I was just told no.

In addition to frustration with management’s lack of receptivity to her requests for safety equipment, participant 3 describes the spatial dimension of COVID-19 risk felt by IPOs who were still required to work in prison. Due to institutional efforts to mitigate the risk of COVID-19 transmission, participants had to adapt the spaces of their face-to-face meetings with prisoners to accommodate physical distancing measures and worried if the spaces were disinfected or if the prisoners were maintaining hygiene standards appropriate for COVID-19 (e.g., handwashing and PPE). Notably, given limited suitable meeting spaces within many prisons, these spatial arrangements meant that IPOs were to share larger meeting spaces with other staff. Participant 6 described the convergence of risks from poor cleanliness and heavily used rooms, stating:

Right now they’re not even allowing us to page inmates or see them in our offices, so they have us kind of running around the jail to these disgusting filthy temporary offices that we share with [staff from] healthcare and programs and psychology. And it’s been a little bit of a nightmare.

As participant 6’s words indicate, COVID-19 made physical spaces in prison take on an additional risk dimension while carrying out their occupational duties—that of contagion in closed spaces. Further, IPOs, like participant 6, expressed frustrations at how hastily implemented safety protocols, such as not using normal offices for meetings, created new health risks. These spatial adaptations created challenges for completing their job duties. Many participants described their meetings with prisoners being rushed, due to staff demand for available interview space, or occurring in spaces that were not conducive to meaningful conversation. IPOs attempting to hold telephone meetings while working off-site had to consider how the new spatial arrangements in prison were affecting the prisoners on their caseload. For example, Participant 79 described the challenges of interviewing a prisoner who could not access a private space to speak on the telephone:

Not all sites are set up for private conversation. Like, [it is a] difficulty if you need to talk about sex offences for offenders. Many of my offenders [on my caseload] are using a telephone that is right outside the barrier, like, right off the range where the inmates live and the inmates are doing their laundry. They’re doing a bunch of laundry, cooking, taking showers, [and] they’re within earshot. So my sex offenders, I’m not fully able to get to the bottom of it, ’cause I’ll ask a question [and] he has to say ‘yes’ [or] ‘no’ [to maintain privacy]. So I’m not going to get a real qualitative [answer] out of the offender.

Spatial restrictions, beyond an inconvenience, impinged on IPOs’ ability to provide meaningful support. Further, in some instances, IPOs found that telephone conversations put clients at risks and they were left trying to mitigate the increased risk to their client posed by exposing the nature of the offence (see Ricciardelli and Moir, 2013; Ricciardelli and Spencer, 2017). In response, IPOs felt hampered in their ability to perform their occupational responsibilities and instead had to find roundabout ways to ask pertinent questions without compromising the safety, security, and confidentiality of their clients.

IPOs also expressed the view that the behavior of prisoners, including inconsistent adoption of mask wearing or physical distancing, created additional risks while they were on-site. As participant 12 explained, there was a mixed reaction to the risk of COVID-19 transmission by prisoners at her place of work:

You see them walking around, no masks, you know, hugging guys and fist bumping them, and acting like it’s normal. They don’t even realize. And then you see others that are definitely taking it more serious, and I think they’re worried about their loved ones out there. And then you see some that are definitely isolating themselves, and I think having those same challenges that people in the community are having about keeping good mental health and not becoming basically a hermit…. [But] I find that majority of the inmates are acting like it’s still just the pandemic has not happened.

In these words, participant 12 identifies prisoners’ behavior as a potential risk for IPOs, while also noting that the mental health challenges caused by increased social isolation are compounding prisoners’ already difficult conditions of confinement. Perceived health risks were not limited to prisoners and extended to IPOs’ coworkers:

There’s also the mask issue. Like, most people are wearing them, but there’s been a lot of instances where like you know, correctional officers aren’t wearing them or inmates are coming out for interviews and they don’t have them. So it’s constantly the need to be like ‘hey, where’s your mask? You need to put your mask on.’ So that part that’s kind of annoying cause we’re all grown-ups and it’s a simple task of putting a mask on….. When I have to remind the staff like constantly like hey, wear your mask or when it’s a correctional officer, like we’re not on the same level either, so it makes for awkward conversations. (participant 40)

As participant 12 and participant 40’s statements show, the context of a pandemic created new risk for frontline correctional workers who feared that prisoners and staff alike may not follow public health protocols and thus be vectors for COVID-19 transmission. Participant 40’s words demonstrate that, in the context of COVID-19, some IPOs perceived *both* prison staff and prisoners as potential risks to their health. Noting that she will regularly call out colleagues and prisoners who are not wearing masks, participant 40 also identifies self-advocacy as a key risk mitigation strategy adopted by IPOs. IPOs asserted a level of control over the risks posed by COVID-19 by confronting what they felt were inadequate safety protocols. Participant 29 stated that, early in pandemic, she questioned her management team on their insistence on holding in-person meeting:

At the beginning of the pandemic, CSC was definitely not taking it seriously. We were mandated to attend a meeting at some point in March, and I put my hand up [and asked] ‘why are we mandated to attend a meeting in [the institution] when its unsafe to do so?’ So they were slow to respond. But I do think that now with the masks and the sanitizer, and there’s lots of protocols in place now, which I think are good, and extra screening for visitors, extra screening for staff, I think that CSC has done a good job trying to manage the risks.

At the onset of the pandemic, all organizations and governments struggled to establish protocols in line with public health guidelines. In Participant 29’s statement, she explains that, facing an unnecessarily risky situation in the early days of the pandemic, she was willing to challenge management on their decisions and to advocate for her concerns. Participant 29 goes on to explain that, ultimately, the CSC implemented various safety protocols to minimize the risks of COVID-19 transmission, satisfying her concerns. Participant 44 similarly identified what she felt was an inadequate organizational response to the risk of COVID-19 in the early days of the pandemic, and noted that she had to develop her own risk mitigation strategies in response:

Until we had official direction from upper management, everybody was kind of using their own personal discretion as far as, like, how to maintain social distance and just, like, the little things like, well what does that mean when you’re interviewing an offender? … There was this added level of kind of confusion and stress as to, like, ‘what are we supposed to be doing here, like what’s our plan of attack?’ Because I’m interpreting it this way, but he clearly [isn’t]. We hadn’t been given direction from anybody in upper management yet, so yeah, there was just a lot of stress around the confusion of to how we’re supposed to be doing business in the first couple months, and like I said, every day you walked into the institution going ‘what are we doing today and how is this going to look today?” And you didn’t know.

In her narration, participant 44 articulates a view that the CSC’s initial response to the pandemic did not provide staff with clear guidance on reducing risks and thus created additional stresses for IPOs who were concerned about infection. Typical of other IPOs, participant 44 navigated these heightened perceptions of risk by attempting to exert control by minimizing close physical contact with people in the institutional spaces she worked. The ability of IPOs to reconstruct their workspace to mitigate risk was constrained or enabled by the built environment of the institution. As noted, many IPOs were forced to use shared office spaces that they felt were not adequately cleaned, some participants worked in institutions that permitted more spatial flexibility for them to conduct meetings with prisoners. As participant 35 explained:

We have a portable trailer so they only can come in the very front, [and] there’s a tiny, tiny waiting area with plexi-glass. So we can talk to them through that with our masks on and their masks on. And you know, sign paperwork and things like that. So I’ve also utilized that little front area. But I’ve also utilized—we have a gazebo in the main compound so you’re able to easily social distance in the gazebo. You’re outside. Still masks on. And its summer so actually a lot of the inmates that I’ve been counselling out there enjoy it because we’re outside.

Here, participant 35 highlights how the space of the prison enabled her to meet prisoners in settings that reduced the likelihood of transmission and, thus, minimized her concerns about COVID-19. Like other participants, she understood her job to carry increased risks due to COVID-19 and developed strategies to navigate her new work environment.

## Discussion

In the current article, we demonstrate how the COVID-19 pandemic shaped IPOs’ understandings of occupational stressors and risk (and need). The imposition of telework improved some participants’ productivity, which aligns with literature suggesting that remote work arrangements may contribute to increased employee productivity (e.g., Martin and MacDonnell 2012; Nakrošienė, Bučiūnienė, and Goštautaitė, 2019). Clearly, the removal of IPOs from the chaos of the prison environment increased IPOs’ ability to focus on writing reports and applications when working at home; as such, their productivity, if measured by increases in written outputs, increased. However, complicating their experiences was the overlap of work and personal space—work came home and home was work. Thus, boundaries, to some necessary for maintaining work–life balance, dissolved in the pandemic—thus exacerbating pre-existing challenges faced by PPOs of work negatively impacting their home life (Morran, 2008; Westaby et al., 2016). Moreover, IPOs’ productivity, in terms of building relationships with and meeting the needs of their clients, was hampered by their time away from the institution. Their inability to meet regularly with prisoners face-to-face disrupted IPOs’ case management duties. Thus, a hybrid model that includes working from home persists post or during COVID-19, warrants efforts to create differentiation between work and home for those necessitating such boundaries.

Staffing reductions also created new stresses for participants, as they had to juggle other IPOs’ caseloads when they were on-site while simultaneously trying to assist prisoners who were unable to access programs or meet with other staff on their case management teams. Moreover, and an area in need for further research, IPOs remained one of the only supports available to prisoners in federal institutions. Thus, when on-site during COVID-19, IPOs were also managing the psychological and physical health concerns of prisoners—they were the support for persons whose programming, and often connection to the outside world (e.g., visits), were largely cut off. Moreover, IPOs were providing supports and responding to needs of prisoners with whom they had little relationship because they were not on their caseload. The additional stress of being an essential service provider to a vulnerable population requires in-depth investigation. It is critical to keep the essential service provider population healthy, including PPOs, and it is possible that their well-being may dissipate given the long-term stresses associated with essential service provision during the pandemic; as such, correctional organizations should implement additional mental health services and supports that continue beyond the pandemic, in order to support the well-being of their staff during and after the challenges of COVID-19.

Layered on these stresses for many IPOs was an increase in parole applications or exceptional releases, the inspiration for which they identified as government calls for and media reports on decarceration. Despite the fact that very few pandemic-related applications were successful, some IPOs found that decarceration significantly increased their workloads. The challenge here also stemmed from how IPOs also had to manage the dismay among the many prisoners who were hoping, or were made to hope, for early release—another area warranting future research. To remedy some of the workload challenges faced by IPOs, and the prisoner responses they must navigate, greater education on criteria determining eligibility for exceptional release or parole during COVID-19 needs to be provided to prisoners. Such clarification and education may help manage expectations among prisoners regarding their eligibility as well as the workloads of IPOs.

IPOs, particularly those still working inside correctional institutions, faced added stresses due to new or exacerbated risk as informed by COVID-19. Specifically, participants identified inadequate safety protocols, spatial arrangements, and the behavior of prisoners as primary sources of risk to their health during the pandemic’s first wave. Weaved throughout descriptions of these perceived risks was frustration from IPOs that their safety concerns were not being sufficiently addressed by management or that protocols were not being consistently implemented. IPOs adopted several strategies in response to these new and heightened risks to their health and safety, including self-advocacy and rearranging their spaces of work. As the pandemic progressed, IPOs tended to report stricter adherence to public health measures in institutions, although challenges remained, for instance, around mask or sanitizer use. Underpinning challenges appear to be communication hiccups that impede necessary pandemic-related information mobilization, a factor requiring consideration in future studies and warranting direct attention from correctional services. Indeed, clear and direct communication from management about health and safety protocols with explicit instructions on consistent and adequate adherence could prove fruitful for protecting the health and safety of all those within prisons. Moreover, directives need to be implemented consistently and without exception to keep the virus at bay in prisons.

COVID-19 did and continues to create new workplace risks, prisoner and staff needs, and occupational challenges for IPOs. Participants explained how COVID-19 changed their experiences of their workspaces and forced them to develop strategies to maintain their safety. IPOs report that some prisoners, coworkers, and management were not taking the virus seriously. In response, an increased health vigilance became required, beyond that which already existed given infectious disease are always of concern in prisons (Hartley et al., 2013) and safety vigilance underpins all prison work or living (Ricciardelli, 2014, 2019). Indeed, institutional correctional work is riddled with the potentiality to be exposed or victim to violence, including potentially psychologically traumatic events—potentialities that are omnipresent—and create a need to engage in behaviors that provide protection among all living and working in prison spaces.

Overall, laced throughout participants’ words was a perception that organizational responses to COVID-19 were limiting their ability to perform their job duties and provide a high level of support to prisoners on their caseload. Thus, the associated new challenges and processes in place due to the pandemic, although necessary for public and institutional health, created barriers to care and support provision for prisoners, both those on a select IPO’s caseload and those on the caseload of other IPOs. The pandemic reduced contact between IPOs and prisoners, and also created new spaces for disappointment and frustration (e.g., unsuccessful applications for early release/parole and elimination of visitation) for prisoners that fell to IPOs to navigate—IPOs who were already struggling to provide supports for prisoners. Thus, practices need to be implemented that ensure IPOs are able to maintain their contact with those on their caseload, including if a hybrid (e.g., work from home part-time) model is to be implemented moving forward.

Our study nonetheless has limitations. As noted, we conducted interviews with IPOs after the first, but before the future waves of the COVID-19 pandemic in Canada. Moreover, participants self-selected to be interviewed and the online process for confirming interviews (as well as the additional administrative load tied to submitting demographic information and consent forms) was likely a deterrent for many potential participants. Indeed, we struggled to keep pace with the speed with which interviews were requested by study participants, which is also a study limitation. Our results may not be generalizable beyond our sample. Despite these limitations, our study provides rich insights into the experiences of IPOs grappling with new or heightened challenges and occupational stressors generated by the onset of the COVID-19 pandemic. As such, the current article, and the recommendations offered herein, provide a foundation for better understanding and redressing the occupational stressors and risks faced by IPOs during and following the pandemic.

## References

[bibr1-20662203211056487] Armstrong GS Atkin-Plunk CA Wells J (2015) The relationship between work-family conflict, correctional officer job stress, and job satisfaction. Criminal Justice and Behavior 42(10): 1066–1082.

[bibr2-20662203211056487] Arnold H (2017) The psychological and emotional effects of prison on prison staff. In: Ireland J Ireland C Fisher F, et al. (eds) The Routledge International Handbook of Forensic Psychology in Secure Settings. London, UK: Routledge, pp. 283–299.

[bibr3-20662203211056487] Barnert E Ahalt C Williams B (2020) Prisons: amplifiers of the COVID-19 pandemic hiding in plain sight. American Journal of Public Health 110(7): 964–966. 32407126 10.2105/AJPH.2020.305713 PMC7287517

[bibr4-20662203211056487] Brennan PK (2020) Responses taken to mitigate COVID-19 in prisons in England and Wales. Victims & Offenders 15(7–8): 1215–1233.

[bibr5-20662203211056487] Burki T (2020) Prisons are “in no way equipped” to deal with COVID-19. Lancet 395(10234): 1411–1412. 32359457 10.1016/S0140-6736(20)30984-3 PMC7252088

[bibr6-20662203211056487] Carleton RN Afifi TO Taillieu T, et al. (2019) Exposures to potentially traumatic events among public safety personnel in Canada. Canadian Journal of Behavioural Science/Revue Canadienne Des Sciences du Comportement 51(1): 37–52.

[bibr7-20662203211056487] Carleton RN Afifi TO Taillieu T, et al. (2020) Assessing the relative impact of diverse stressors among public safety personnel. International Journal of Environmental Research and Public Health 17(4): 1234. 32075062 10.3390/ijerph17041234 PMC7068554

[bibr8-20662203211056487] CBC News (2021) Tracking the Coronavirus. Toronto, Canada: CBC News. https://newsinteractives.cbc.ca/coronavirustracker/(accessed 15 June 2021).

[bibr9-20662203211056487] Charmaz K (2014) Constructing Grounded Theory. 2nd edition. Thousand Oaks, CA: SAGE.

[bibr10-20662203211056487] Correctional Service of Canada (2019a) CSC Statistics - Key Facts and Figures. Ottawa, Canada: Correctional Service of Canada. Available at: https://www.csc-scc.gc.ca/publications/005007-3024-en.shtml (accessed 20 May 2021.

[bibr11-20662203211056487] Correctional Service of Canada (2019b) The Correctional Process. Ottawa, Canada: Correctional Service of Canada. Available at https://www.csc-scc.gc.ca/publications/005007-3011-en.shtml?wbdisable=true (accessed 25 May 2021).

[bibr12-20662203211056487] Correctional Service of Canada (2019c) Types of Release. Ottawa, Canada: Correctional Service of Canada. Available at: https://www.csc-scc.gc.ca/parole/002007-0002-en.shtml (accessed 25 August 2021).

[bibr13-20662203211056487] Correctional Service of Canada (2021) Testing of inmates in federal correctional institutions for COVID-19. Available at: https://www.csc-scc.gc.ca/001/006/001006-1014-en.shtml (accessed 14 April 2021).

[bibr14-20662203211056487] DeMichele M Payne BK (2007) Probation and parole officers speak out—caseload and workload allocation. Federal Probation 71(3): 30–35.

[bibr15-20662203211056487] Duxbury L Higgins C Halinski M (2015) Identifying the Antecedents of Work-Role Overload in Police Organizations. Criminal Justice and Behavior 42(4): 361–381.

[bibr58-20662203211056487] Farrow K (2004) Still committed after all these years? Morale in the modern-day probation service. Probation Journal 51(3): 206–220. DOI: 10.1177/0264550504045898.

[bibr16-20662203211056487] Finn P Kuck S (2005) Stress Among Probation and Parole Officers and what Can Be Done about it. Washington DC: National Institute of Justice.

[bibr17-20662203211056487] Glaser BG Strauss AL (1967) The Discovery of Grounded Theory: Strategies for Qualitative Research. Chicago, IL: Aldine Publishing.

[bibr18-20662203211056487] Harris K (2020) Bill Blair Asks Prison, Parole Heads to Consider Releasing Some Inmates to Stop Spread of COVID-19. Toronto, Canada: CBC News. 31 March 2020. Available at: https://www.cbc.ca/news/politics/prison-covid19-csc-release-1.5516065 (accessed 10 May 2021).

[bibr19-20662203211056487] Hartley DJ Davila MA Marquart JW, et al. (2013) Fear is a disease: the impact of fear and exposure to infectious disease on correctional officer job stress and satisfaction. American Journal of Criminal Justice 38(2): 323–340.

[bibr20-20662203211056487] Kothari R Forrester A Greenberg N, et al. (2020) COVID-19 and prisons: providing mental health care for people in prison, minimising moral injury and psychological distress in mental health staff. Medicine, Science and the Law 60(3): 165–168. 32462971 10.1177/0025802420929799

[bibr21-20662203211056487] Kinner SA Young JT Snow K, et al. (2020) Prisons and custodial settings are part of a comprehensive response to COVID-19. The Lancet Public Health 5(4): e188–e189. 32197116 10.1016/S2468-2667(20)30058-X PMC7103922

[bibr22-20662203211056487] Lai C-C Wang J-H Ko W-C, et al. (2020) COVID-19 in long-term care facilities: an upcoming threat that cannot be ignored. Journal of Microbiology, Immunology and Infection 53(3): 444–446. 10.1016/j.jmii.2020.04.008 PMC7153522 32303483

[bibr23-20662203211056487] Lambert EG Hogan NL (2018) Correctional staff: the issue of job stress. In: Ternes M Magaletta P Patry M (eds) The Practice of Correctional Psychology. Berlin, Germany: Springer, pp. 259–281.

[bibr24-20662203211056487] Lewis KR Lewis LS Garby TM (2013) Surviving the trenches: the personal impact of the job on probation officers. American Journal of Criminal Justice 38(1): 67–84.

[bibr25-20662203211056487] Lindström M Ahlstrand E Kärrholm J (2020) Sweden’s response to the COVID-19 outbreak. Victims & Offenders 15(7–8): 1203–1214.

[bibr26-20662203211056487] Marcum CD (2020) American corrections system response to COVID-19: an examination of the procedures and policies used in Spring 2020. American Journal of Criminal Justice 45: 759–768. 32837155 10.1007/s12103-020-09535-3 PMC7275927

[bibr27-20662203211056487] Harker Martin B MacDonnell R (2012) Is telework effective for organizations? Management Research Review 35(7): 602–616.

[bibr28-20662203211056487] Mealer M Jones RN J (2014) Methodological and ethical issues related to qualitative telephone interviews on sensitive topics. Nurse Researcher 21(4): 32–37. 10.7748/nr2014.03.21.4.32.e1229 24673351

[bibr57-20662203211056487] Morran D (2008) Firing up and burning out: The personal and professional impact of working in domestic violence offender programmes. Probation Journal 55(2): 139–152. DOI: 10.1177/0264550508090272.

[bibr29-20662203211056487] Nakrošienė A Bučiūnienė I Goštautaitė B (2019) Working from home: characteristics and outcomes of telework. International Journal of Manpower 40(1): 87–101.

[bibr30-20662203211056487] Norman M Ricciardelli R (2021) Operational and organisational stressors in community correctional work: insights from probation and parole officers in Ontario, Canada. Probation Journal. Epub ahead of print 3 February 2021. DOI: 0264550520984253. 10.1177/0264550520984253 PMC8939856 35340787

[bibr31-20662203211056487] Novick G (2008) Is there a bias against telephone interviews in qualitative research? Research in Nursing & Health 31(4): 391–398. 18203128 10.1002/nur.20259 PMC3238794

[bibr32-20662203211056487] Nowotny KM Seide K Brinkley-Rubinstein L (2021) Risk of COVID-19 infection among prison staff in the United States. BMC Public Health 21(1): 1036–1038. 34078350 10.1186/s12889-021-11077-0 PMC8170443

[bibr33-20662203211056487] O'Donnell C Stephens C (2001) The impact of organisational, social environmental and job content stressors on the work related strains of probation officers. Australian & New Zealand Journal of Criminology 34(2): 193–203.

[bibr34-20662203211056487] Office of the Correctional Investigator (2021) Third COVID-19 Status Update (February 23). Ottawa, Canada: Office of the Correctional Investigator. Available at: https://www.oci-bec.gc.ca/cnt/rpt/pdf/oth-aut/oth-aut20210223-eng.pdf (accessed 14 April 2021).

[bibr35-20662203211056487] Page J Robertson N (2021) Extent and predictors of work-related distress in community correction officers: a systematic review. Psychiatry, Psychology and Law: 1–28. Epub ahead of print 7 April 2021. DOI: 10.1080/13218719.2021.1894259. PMC9225775 35755153

[bibr36-20662203211056487] Parole Board of Canada (2021) COVID-19 and the Parole Board of Canada. Ottawa, Canada: Parole Board of Canada. Available at: https://www.canada.ca/en/parole-board/services/coronavirus-covid-19.html (accessed 10 May 2021).

[bibr37-20662203211056487] Persson A Svensson K (2011) Signs of resistance? Swedish probation officers’ attitudes towards risk assessments. European Journal of Probation 3(3): 95–107.

[bibr38-20662203211056487] Phillips J Westaby C Fowler A (2016) “It’s relentless”. Probation Journal 63(2): 182–192.

[bibr39-20662203211056487] Public Safety Canada (2021) COVID-19 Planning for Federal Corrections. Ottawa, Canada: Public Safety Canada. Available at: https://www.securitepublique.gc.ca/cnt/trnsprnc/brfng-mtrls/prlmntry-bndrs/20200930/019/index-en.aspx?wbdisable=true (accessed 25 May 2021).

[bibr40-20662203211056487] Rhineberger-Dunn G Mack KY Baker KM (2016) Secondary trauma among community corrections staff: an exploratory study. Journal of Offender Rehabilitation 55(5): 293–307.

[bibr41-20662203211056487] Ricciardelli R (2014) Surviving Incarceration: Inside Canadian Prisons. Waterloo, Canada: Wilfrid Laurier University Press.

[bibr42-20662203211056487] Ricciardelli R (2018) “Risk it out, risk it out”: occupational and organizational stresses in rural policing. Police Quarterly 21(4): 415–439.

[bibr43-20662203211056487] Ricciardelli R (2019) Also Serving Time: Canada’s Provincial and Territorial Correctional Officers. Toronto, Canada: University of Toronto Press.

[bibr44-20662203211056487] Ricciardelli R Bucerius S Tetrault J, et al. (2021) Correctional services during and beyond COVID-19. FACETS 6(1): 490–516.

[bibr45-20662203211056487] Ricciardelli R Clow KA White P (2010) Investigating hegemonic masculinity: Portrayals of Masculinity in Men’s Lifestyle Magazines. Sex Roles 63(1–2): 64–78.

[bibr46-20662203211056487] Ricciardelli R Moir M (2013) Stigmatized among the stigmatized: sex offenders in Canadian penitentiaries. Canadian Journal of Criminology and Criminal Justice 55(3): 353–386.

[bibr47-20662203211056487] Ricciardelli R Spencer DC (2017) Violence, Sex Offenders, and Corrections. New York, NY: Routledge.

[bibr48-20662203211056487] Ryan C Sabourin H Ali A (2020) Applying an Indigenous and gender-based lens to the exploration of public health and human rights implications of COVID-19 in Canadian correctional facilities. Canadian Journal of Public Health 111(6): 971–974. 33074479 10.17269/s41997-020-00426-y PMC7571293

[bibr49-20662203211056487] Slate RN Johnson WW (2013) Stressors experienced by state and federal probation officers. In: Miller MK Bornstein BH (eds) Stress, Trauma, and Wellbeing in the Legal System. New York, NY: Oxford University Press, pp. 197–216.

[bibr50-20662203211056487] Stewart A Cossar R Stoové M (2020) The response to COVID-19 in prisons must consider the broader mental health impacts for people in prison. Australian & New Zealand Journal of Psychiatry 54(12): 1227–1228. 32571078 10.1177/0004867420937806 PMC7312101

[bibr51-20662203211056487] Strauss J Corbin A (1990) Basics of Qualitative Research: Techniques and Procedures for Developing Grounded Theory. Thousand Oaks, CA: SAGE.

[bibr52-20662203211056487] Testoni I Francioli G Biancalani G, et al. (2021) Hardships in Italian prisons during the COVID-19 emergency: the experience of healthcare personnel. Frontiers in Psychology 12: 1–8. 10.3389/fpsyg.2021.619687 PMC7890194 33613396

[bibr53-20662203211056487] Tsai J Wilson M (2020) COVID-19: a potential public health problem for homeless populations. The Lancet Public Health 5(4): e186–e187. 32171054 10.1016/S2468-2667(20)30053-0 PMC7104053

[bibr54-20662203211056487] Weaver B Barry M (2014) Managing high risk offenders in the community: compliance, cooperation and consent in a climate of concern. European Journal of Probation 6(3): 278–295.

[bibr55-20662203211056487] Westaby C Phillips J Fowler A (2016) Spillover and work-family conflict in probation practice: managing the boundary between work and home life. European Journal of Probation 8(3): 113–127.

[bibr56-20662203211056487] Yang H Thompson JR (2020) Fighting Covid-19 outbreaks in prisons. BMJ (Clinical Research ed.) 369(m1362): m1362. 10.1136/bmj.m1362 32241756

